# Sepsis in the Neurologic Intensive Care Unit: Epidemiology and Outcome

**DOI:** 10.14740/jocmr1935w

**Published:** 2014-10-16

**Authors:** Farid Sadaka, Margaret A Cytron, Kimberly Fowler, Victoria M Javaux, Jacklyn O’Brien

**Affiliations:** a Mercy Hospital St. Louis; St. Louis University, MO 63141, USA

**Keywords:** Sepsis, Epidemiology, Neurologic intensive care unit, Outcome

## Abstract

**Background:**

Sepsis is a major contributor to mortality in patients admitted to a general intensive care unit (ICU). Early recognition and treatment of sepsis is key in improving outcomes. The epidemiology and outcome of sepsis in neurologic ICU (NeuroICU) has not been evaluated.

**Methods:**

We retrospectively identified all patients admitted to our 16-bed NeuroICU between June 2009 and December 2013 using the acute physiologic and chronic health evaluation (APACHE) outcomes database. We excluded patients admitted with an infection, such as meningitis, encephalitis, brain or spinal abscess, or with any other infection. We compared NeuroICU patients who did to NeuroICU patients who did not develop sepsis after ICU admission. The diagnosis of sepsis was based on the SCCM/ACCP consensus conference definition.

**Results:**

There were a total of 2,025 patients, out of which 29 patients (1.4%) developed sepsis. Patients who developed sepsis had a trend towards older age (67 ± 13 vs. 61 ± 11 years, P = 0.07), a trend towards more male gender (69.0% vs. 51.5%, P = 0.07), significantly higher APACHE III scores (58 ± 17 vs. 43 ± 21, P = 0.0001), and significantly higher acute physiologic scores (APS) (43 ± 16 vs. 32 ± 18, P = 0.001) than patients who did not develop sepsis. Patients who developed sepsis had higher ICU mortality (41.4% vs. 5.1%, odds ratio (OR) = 13.1; 95% confidence interval (CI), 6.1 - 28.2, P < 0.0001), and higher hospital mortality (44.8% vs. 8.2%, OR = 9.0; 95% CI, 4.3 - 19.0, P < 0.0001).

**Conclusions:**

Sepsis developed in 1.4% of patients admitted to a NeuroICU. Predictors of sepsis development were comorbidities and worsening acute physiologic variables. Patients who developed sepsis had significantly higher mortality. Vigilance to development of sepsis in NeuroICU is paramount, especially in this era when early recognition and intervention of sepsis significantly improves outcomes.

## Introduction

In the United States, approximately 750,000 cases of sepsis occur each year, of which at least 225,000 are fatal. If it also causes organ dysfunction, the diagnosis is severe sepsis. If severe sepsis is accompanied with tissue hypoperfusion, the diagnosis is septic shock. Organ failure occurs in about one-third of patients with sepsis, and severe sepsis is associated with an estimated mortality rate of 30-50%. There is wide variation in the incidence of sepsis and severe sepsis in the general intensive care unit (ICU) setting, with reported rates ranging from 20% to 80%, and reported mortality of 20% to 50%. Septic shock, defined as a state of acute circulatory failure characterized by persistent hypotension unexplained by other causes, despite adequate fluid resuscitation, affects between 10% and 30% of patients managed in the ICU, and its incidence is increasing. Mortality from septic shock in the ICU is estimated to range between 45% and 63% in observational studies [1-7]. However, epidemiology of sepsis comes mainly from general medical and surgical ICUs. Epidemiology and outcome of sepsis in neurologic ICUs (NeuroICUs) has not yet been reported.

The primary objective of this study was to report the epidemiology and outcomes of sepsis in the NeuroICU.

## Methods

We retrospectively identified all patients admitted to our NeuroICU between June 2009 and December 2013 using the acute physiologic and chronic health evaluation (APACHE) outcomes database. Our 16-bed NeuroICU is staffed by intensivists (board certified by the American Board of Internal Medicine in Internal Medicine and Critical Care Medicine and certified by the United Council of Neurologic Subspecialties in Neurocritical care) 24 h/day. APACHE outcomes database is a free, web-based offering from Cerner Corporation that provides users the ability to calculate and report on outcomes data based upon the APACHE IV predictions available in the public domain. The system may then be used either as an on-line calculator to quickly obtain scores and predictions for individual patients or subsets of patients on an as needed basis, or as a severity-adjusted outcomes measurement system for an individual ICU (or group of ICUs) to assess quality of care and identify opportunities for improvement. We excluded patients admitted with an infection, such as meningitis, encephalitis, brain or spinal abscess, or with any other infection. We compared NeuroICU patients who did to NeuroICU patients who did not develop sepsis after ICU admission. The diagnosis of sepsis is based on surviving sepsis campaign (SSC) definition [8]. It is not standard in our institution to start prophylactic antibiotics. Antibiotics are prescribed when patient is diagnosed with or suspected to have an infection or sepsis. Data were entered using a software program that included computerized pick lists and automated calculation of physiological means and gradients and error checking. Patient identifiers were removed from the database, and informed consent was waived by our institutional review board.

## Results

**Figure 1:**
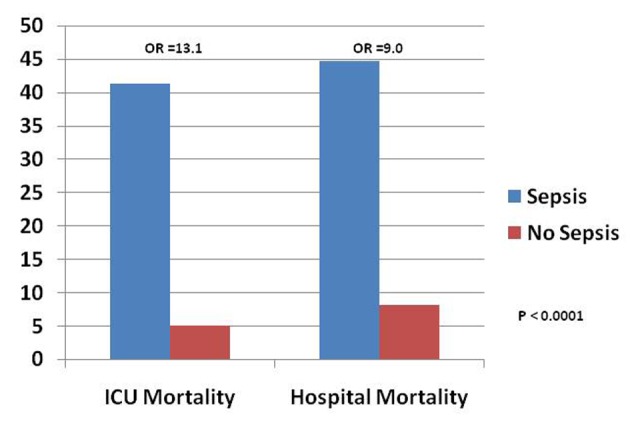
Outcomes of patients in NeuroICU who did and did not develop sepsis.

There were a total of 2,025 patients, out of which 29 patients (1.4%) developed sepsis. Patients who developed sepsis had a trend towards older age (67 ± 13 vs. 61 ± 11 years, P = 0.07), a trend towards more male gender (69.0% vs. 51.5%, P = 0.07), significantly higher APACHE III scores (58 ± 17 vs. 43 ± 21, P = 0.0001), and significantly higher acute physiologic scores (APS) (43 ± 16 vs. 32 ± 18, P = 0.001) than patients who did not develop sepsis (Table 1). Patients who developed sepsis had higher ICU mortality (41.4% vs. 5.1%, odds ratio (OR) = 13.1; 95% confidence interval (CI), 6.1 - 28.2, P < 0.0001), and higher hospital mortality (44.8% vs. 8.2%, OR = 9.0; 95% CI, 4.3 - 19.0, P < 0.0001) (Table 1, Fig. 1).

**Table 1:** Characteristics and Outcomes of Patients in the NeuroICU Who Did and Did Not Develop Sepsis

	Sepsis	No sepsis	P value
n, %	29 (1.4)	1,996 (98.6)	
Age, years (SD)	67 (13)	61 (11)	0.07
Gender, male, %	69	52	0.07
APACHE III score (SD)	58 (17)	43 (21)	0.0001
APS score (SD)	43 (16)	32 (18)	0.001
Top three diagnoses, n (%)	TBI, 5 (17.2)	TBI, 316 (15.8)	
	ICH, 5 (17.2)	Ischemic stroke, 290 (14.5)	
	Encephalopathy, 5 (17.2)	Seizures, 190 (9.5)	
ICU mortality, n (%)	12 (41.4)	102 (5.1)	< 0.0001
Hospital mortality, n (%)	13 (44.8)	164 (8.2)	< 0.0001

APACHE: acute physiologic and chronic health evaluation; APS: acute physiologic score; ICU: intensive care unit; SD: standard deviation; TBI: traumatic brain injury; ICH: intracranial hemorrhage.

## Discussion

In our center, sepsis developed in 1.4% of patients admitted to a NeuroICU. Predictors of sepsis development were comorbidities and worsening acute physiologic variables. Patients who developed sepsis had significantly higher mortality. Sepsis is the number one cause of death in the non-coronary ICU [9]. Timely, aggressive, and efficient recognition and management of patients with sepsis/severe sepsis/septic shock is crucial, particularly with the increasing incidence, costs, and mortality associated with untimely management of these patients.

Dramatic benefit of early goal-directed therapy (EGDT) in a single-center study of patients with severe sepsis/septic shock published by Rivers et al in 2001 created a paradigm shift on how we treat these patients [10]. Since then, guidelines were published by SSC in 2004, 2008, and 2012 [8] in order to guide management of sepsis/severe sepsis/septic shock. In summary, resuscitation parameters have to be accomplished within 6 h of diagnosis in order to achieve improvement in survival [8]. In a very recent study “Protocolized care for early septic shock (ProCESS) trial”, septic shock patients were randomly assigned to one of three groups for 6 h of resuscitation: protocol-based EGDT; protocol-based standard therapy that did not require the placement of a central venous catheter, administration of inotropes, or blood transfusions; or usual care [11]. All three groups in this study had similar outcomes. One important contribution of the ProCESS trial is the evidence it provided regarding the ongoing role of early recognition of sepsis in improving survival. The ProCESS trial showed the paramount positive effect of early recognition of sepsis, early administration of antibiotics, and early adequate volume resuscitation, on outcomes. Despite the low prevalence of sepsis in the NeuroICU (1.4%), the high mortality of patients with sepsis, the importance of early recognition and treatment, and the tremendous improvement in outcomes associated with early recognition and treatment underscore the need for early identification of septic patients in the NeuroICU.

Our study has several limitations. It is a retrospective and a single-center study. However, it encompasses a large cohort of patients, and the outcomes of septic patients in our study are similar to other larger multicenter studies [1-8]. Our NeuroICU may not be representative of other NeuroICUs. Our center is a large university-affiliated hospital with 1,000 beds, primary stroke center and a level I trauma center, which might explain the three top diagnoses in our patient population (Table 1). Hence, epidemiology might be different in other centers that may not be stroke centers, trauma centers, or university-affiliated hospitals.

### Conclusion

Sepsis developed in 1.4% of patients admitted to a NeuroICU. Predictors of sepsis development were comorbidities and worsening acute physiologic variables. Patients who developed sepsis had significantly higher mortality. Vigilance to development of sepsis in NeuroICU is paramount, especially in this era when early recognition and intervention of sepsis significantly improves outcomes. Larger multicenter studies are warranted to more accurately report the epidemiology of sepsis development in NeuroICUs.
